# Climate change impacts on the threatened terrestrial vertebrates of the Pacific Islands

**DOI:** 10.1038/s41598-017-05034-4

**Published:** 2017-07-13

**Authors:** Lalit Kumar, Mahyat Shafapour Tehrany

**Affiliations:** 1 Ecosystem Management, School of Environmental and Rural Science, University of New England, Armidale, NSW 2351 Australia; 2 Geospatial Science, School of Science, RMIT University, Melbourne, VIC, 3000 Australia

## Abstract

The aim of this study was to undertake a broad-scale understanding of the distribution of vulnerable, endangered and critically endangered terrestrial vertebrate species in the Pacific and the assessment of impacts of climate change and sea level rise. 150 critically endangered, endangered and vulnerable terrestrial vertebrates found in 23 countries of the Pacific were linked to island susceptibility to climate change. Out of the 1779 islands making up the 23 countries, 674 of them hosted at least one species from the above categories. 84 of the 150 species are endemic to this study area and many of these occur on islands with high susceptibility to climate change, with many of them occurring on one island only. The species data, together with islands, was overlain with Mean Significant Wave Height (Hs) projections for 2081–2100 under RCP4.5 and 8.5, and further analysed for threat of extinction. A large number of critically endangered and endangered species fall in regions that have the highest Hs projections.

## Introduction

Island communities are often regarded as being at the forefront of impacts from environmental hazards, and more so from climate change. Islands are comparatively small in size, are generally remote, highly volatile and with changing geomorphology, lack connectedness and have a high shoreline to land area^1^, hence are particularly vulnerable to climate change related impacts. Smaller islands are much more vulnerable than larger land masses to environmental changes due to the exposure to surrounding oceans, and the flora and fauna found on them are especially vulnerable due to small resource areas and a lack of opportunity to ‘migrate’ to surrounding areas as climate change has its impacts. While there is a considerable body of research detailing potential climate change related impacts on islands and island communities^2^, there is not much work on climate change related impacts on the flora and fauna found on small island states. Islands are recognised centres of range-restricted species and have high levels of endemism^3^. Information on endemism, species richness and climate change related vulnerabilities are important for the global prioritization of conservation of the affected species^4, 5^, however such information for islands have remained relatively unexplored. More than 20% of the world’s biodiversity is found within the 180,000 islands world-wide^6^. Insular endemics found on islands have generally evolved traits such as reduced or loss of dispersal abilities, including loss of flight in birds and insects and a loss of defensive characteristics^7–9^. Such evolution leads to lower genetic variation and these species are significantly inbred compared to non-endemics^10, 11^. Such characteristics, when combined with the loss of even small amounts of their restricted habitat from climate change related impacts, such as sea level rise, storms and wave action, and the resulting habitat fragmentation makes them highly vulnerable to extinction^12^. Since many of these species are endemic to a few islands only, the extinction of such species from these islands would mean global extinction of those species. The concept of niche width and species diversification^13^ seldom applies to the smaller islands as they have limited land area.

The aim of this study was to undertake a broad-scale understanding of the distribution of vulnerable, endangered and critically endangered terrestrial vertebrate species in the Pacific and the assessment of impacts of climate change and sea level rise. Species vulnerability classes were combined with an island susceptibility categorization (very low, low, medium, high and very high) that was derived from a combination of island lithology, area, shape (roundness) and elevation. A total of 150 terrestrial vertebrate species that were ranked as vulnerable, endangered or critically endangered by the IUCN^14^ were overlaid on an island database with 1779 islands and matched with island susceptibilities (to climate change). The information generated was then overlain on regional scale sea level rise and wave height projections to identify those species that occurred on the fewest islands, on islands with the highest susceptibility categorization, and that fell in the most vulnerable zones as identified by high projected sea level rise and highest wave heights. We have used two approaches for assessing the potential impact of climate change on the vertebrates. Firstly we used island geometry and lithology to develop and index of susceptibility of islands. This does not consider climate impacts. Vertebrates found on more susceptible islands are generally more vulnerable. Secondly we used projected sea level rise and mean significant wave heights to look at the impact of climate change on the islands and identify those islands with high and very susceptible rankings that fell in regions of high projected sea level rise and high mean significant wave heights. Species on these islands are more vulnerable than those on similar islands but falling in lower projected sea level rise regions. The information generated here identifies the most vulnerable terrestrial vertebrate species in the Pacific that need prioritization in terms of conservation efforts.

## Methods

**Figure 1:**
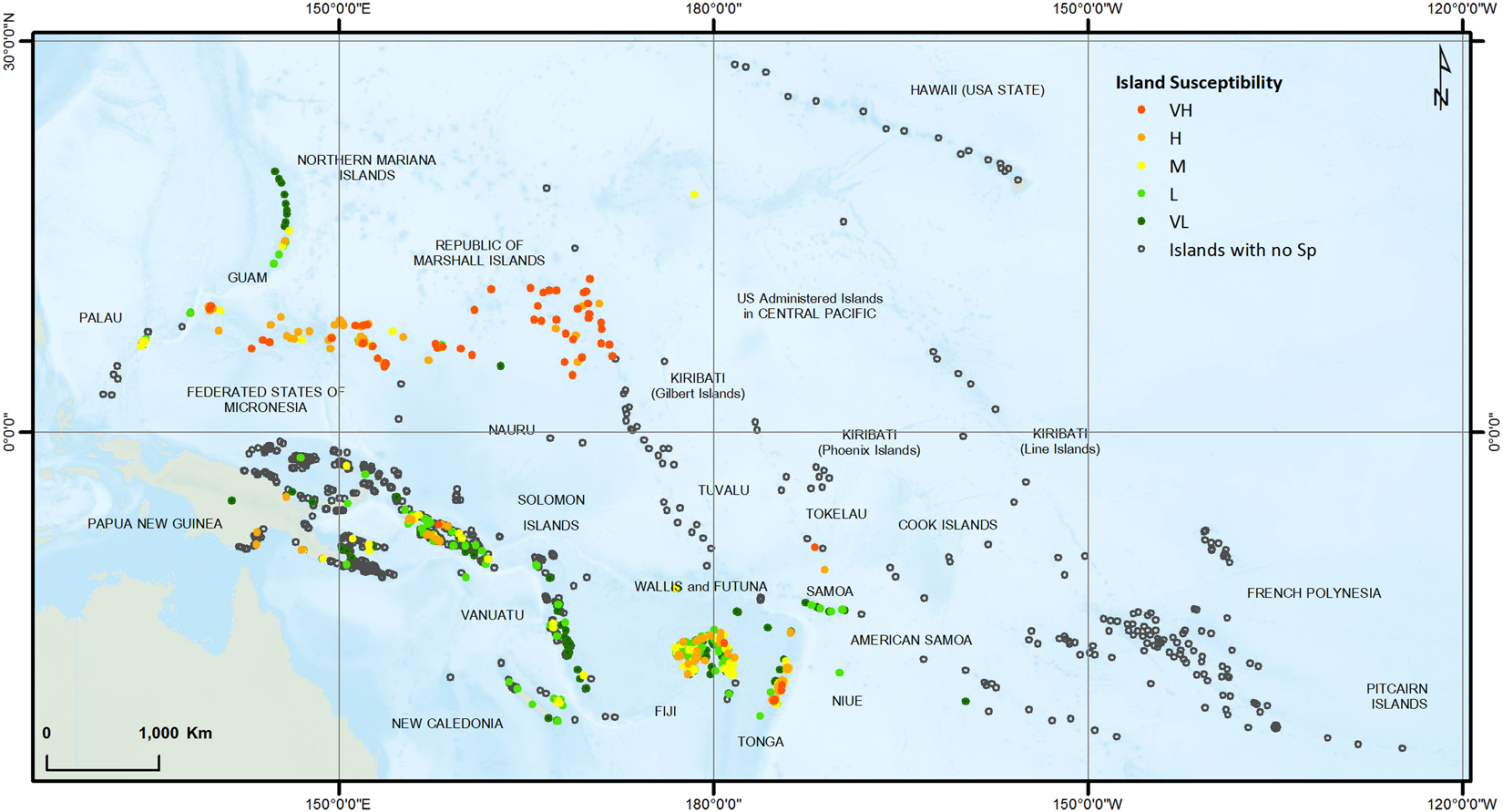
Islands that host at least one vulnerable, endangered or critically endangered terrestrial vertebrate species, with islands susceptibility rankings. (Figure generated using ARCGIS 10.3, http://www.esri.com/).

The region of study for this research was the area bounded by latitudes 30°06′56′N and 27°02′57′S, and longitudes 126°06′56.938′E and 119°17′53.012′W (Fig. 1), so essentially the whole of the Pacific region extending from Palau in the west to the Pitcairn Islands in the east, near the American west coast, excluding Australia, New Zealand, East Timor and the Indonesian Islands. The area of the region is close to 85 million sq km and has over 2000 islands. Nunn, *et al*.^15^ created a database of 1532 islands in this region and categorized each of the islands based on susceptibility to climate change. This database was extended to include another 247 islands, making a total of 1779 islands. The extra islands were added to include countries not covered by the Nunn, *et al*.^15^ report. Island indicative susceptibility index was developed using four island characteristics: lithology, area, maximum elevation and shape (circularity). Lithology, referring to the relative hardness or softness of the dominant rock type and a measure of erodibility, had eight categories: continental, volcanic high, volcanic low, composite high, composite low, limestone high, limestone low and reef islands. Lithology is an important characteristic as it determines the stability or erodibility of an island. A small volcanic island is a lot less likely to be easily eroded by waves compared to a small sandy island, with loose sediments being easily moved around by waves, wind and rain. Species on such islands are naturally more vulnerable. Area, shape (circularity) and maximum elevation provide a three dimensional geometric description of the island. The smaller the island the more susceptible, the higher the island the less susceptible and the less circular the island the more susceptible. The island susceptibility index was developed using readily available or calculable variables, noting that more detailed data for many of the islands is not available. For example, we used maximum elevation as one of the variables as this can be easily obtained from Google Earth for each of the islands. The median elevation may have been a better variable, however fine resolution digital elevation data are not available for most of the islands and hence the mean or median elevations of each of the islands cannot be derived. Details of cutoff values and ranking criteria are given in Nunn, *et al*.^15^. A similar method was applied to the remaining 247 islands that were added to the database. Islands were ranked in susceptibility from very low to very high (very low, low, medium, high, very high), indicating long-term susceptibility of each island to physical change due to climatic and oceanic processes. It should be mentioned here that this is a generalist approach and does not look at the explicit habitats of each of the species. We are saying that, generally, species on a small, low, narrow sandy island are more vulnerable than those on a large, high, round volcanic island; however this will not always be true. Species in coastal areas will be more vulnerable than those inland, however this is a whole-island index and appropriate for a regional scale analysis.

Species vulnerability data was obtained from the IUCN Red list^14^. This database has species names, their vulnerability rankings and distribution detail. All threatened species (vulnerable, endangered and critically endangered) found in the study region and belonging to the taxonomic groups of amphibians, mammals and reptiles (terrestrial vertebrates) were downloaded and marine species excluded. Distribution data was downloaded in the form of polygon vector files. All data was re-projected to the same projection as the island database so that they could be overlain together and relevant information extracted for analysis.

Finally, the extracted islands on which the species occurred, together with their indicative susceptibility values, was overlain on regional scale sea level rise and projected mean significant wave height (Hs) information. This analysis was undertaken to identify those species that occurred on only a few islands with a high susceptibility category, were endemic to the Pacific and would be impacted by relatively higher sea level rise and mean significant wave height. What we are saying here is that, given two islands with exactly the same physical characteristics (same area, maximum elevation, shape and lithology), species on the one impacted by a higher sea level rise and mean significant wave height would be more vulnerable as that island becomes more susceptible under such conditions. For example, if both islands were small, low, narrow and sandy, the one in the projected higher sea level rise region would be more susceptible to change and thus species on that island would generally be more vulnerable. Mean Significant Wave Height (Hs) data was obtained from the South Pacific Applied Geoscience Commission (SOPAC). Two concentration pathways, RCP 4.5 (a medium low emission scenario) and RCP 8.5 (a high emission scenario) were used under the CMIP5 (Coupled Model Intercomparison Project Phase 5) model. The climate models used were CNRM-CM5, HadGEM2-ES, INMCM4 and ACCESS1.0. An ensemble average was obtained for 2081–2100 and the difference between the projected and the historical scenario (1986–2005) was calculated in order to obtain the projected changes in Hs. This projection was on a monthly basis. The data was then imported into ARCGIS and a single layer was created that showed the maximum Hs expected at each pixel during the whole year.

## Results and Discussion

The area of the islands in the database ranged from 0.013 sq km to 35,780 sq km, with the median area being 1.34 sq km, indicating that the islands in the Pacific are predominantly small. The maximum elevation values ranged from 1 m to 4205 m, with the median being 21 m and 42% of islands having a maximum elevation of less that 10 m, indicating that most islands in the Pacific are low lying. Volcanic islands accounted for 39% of the islands, followed by reef islands (36%), limestone (17%), composite (7%) and continental (<1%). In terms of island susceptibility, 12% were ranked very high, 29% as high, 23% as moderate, 23% as low and 13% as very low.

Interrogating the IUCN database resulted in a record of 150 vulnerable, endangered or critically endangered terrestrial vertebrates in the study area. There were 11 amphibians, 67 mammals and 72 reptiles. Among the 150 recorded species, 51, 61 and 38 species belonged to the three categories of vulnerable, endangered, and critically endangered respectively. Table 1 shows the breakdown in terms of taxonomic groups and threat levels.

**Table 1:** Vulnerable, endangered and critically endangered species found in the study site.

	Vulnerable	Endangered	Critically Endangered	**Totals**
Amphibians	10	0	1	11
Mammals	21	28	18	67
Reptiles	20	33	19	72
**Totals**	51	61	38	150

Out of 1779 islands, 674 of them hosted at least one species from the extracted species list. Of these 674 islands, 59 islands had very high susceptibility, 178 had high susceptibility, 152 had medium susceptibility, 171 had low susceptibility, and 114 had very low susceptibility ranking. The remaining 1105 islands did not host any of the vulnerable, endangered or critically endangered terrestrial vertebrates. Of the 674 islands that hosted at least one species, 15 islands hosted 6 or more species (Table S1), 3 islands hosted 5 species, 34 islands hosted 4 species, 143 islands hosted 3 species, 207 islands hosted 2 species and 272 islands hosted 1 species only. The 15 islands that hosted 6 or more species are either Continental, Composite High or Volcanic High islands^15^ and with relatively large areas. In fact these 15 islands together make 84% of the total area covered by the 1779 islands and those 15 islands are the largest of the 1779 in area.

Figure 1 shows the islands that hosted at least one species and islands with no species. Islands that do not host any of the recorded species are presented by empty circles. Moreover, Fig. 1 gives information regarding the islands’ susceptibility ranking. As can be seen, most of the high and very high susceptible islands that host at least one of the species are grouped around each other and placed in the south-southwest, southwest and northwest regions of the Pacific. One of the reasons for this is that this is where we have the larger islands of the Pacific, such as Viti Levu and Vanua Levu of the Fiji group, Espiritu Santo and Malekula of Vanuatu, and La Grande Terre of New Caledonia. Most of these are volcanic or high limestone islands, with relatively large areas. Guam and the Northern Mariana Islands are an exception to this.

**Figure 2:**
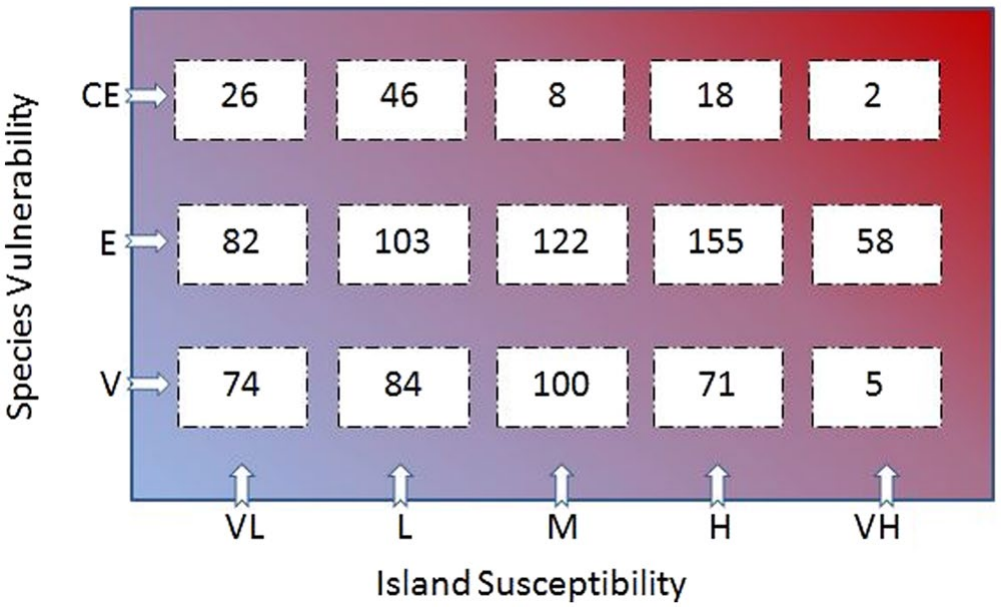
Number of islands that hosted species from different categories.

Figure 2 shows the number of islands with different susceptibility rankings that hosted species from different categories of critically endangered (CE), endangered (E) and vulnerable (V). The red-shaded area shows islands with the highest susceptibility ranking and hosting species under the highest threat levels. For example, 2 islands with very high susceptibility ranking each host at least one critically endangered species.

**Figure 3:**
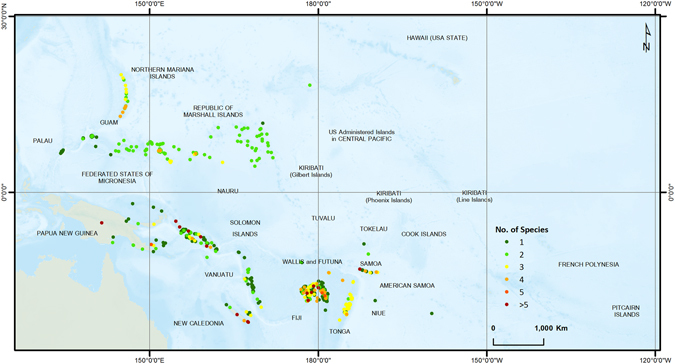
Species types hosted by each of the islands. (Figure generated using ARCGIS 10.3, http://www.esri.com/).

Figure 3 illustrates the spatial distribution of the species (from all three categories of mammals, reptiles and amphibians) in the islands without considering their vulnerability condition. Dark green dots represent the islands which host one species only, and dark red dots show the islands with more than five types of species. As can be seen, some islands are host to only one type of species while many islands hosted more than one species. Species found on one or only a few islands are generally more vulnerable to extinction while species found on multiple islands generally have a higher chance of survival. Table 2 summarizes this information. The 59 very high susceptible islands host a total of 12 unique species (those species found on one susceptibility class islands only); 3 of them from the vulnerable class, 8 from endangered class and 1 from the critically endangered class. The 178 islands with high susceptibility ranking that host at least one species are home to 26 unique species, 8 vulnerable, 12 endangered and 6 critically endangered. Critically endangered species that are found on very high or high susceptible islands are *Pteropus insularis (Chuuk flying fox), Brachylophus vitiensis (Fiji crested iguana), Emoia slevini (Mariana Skink), Pteralopex flanneryi (Greater Monkey-faced Bat), Solomys ponceleti (Poncelet’s Giant Rat)* and *Spilocuscus rufoniger (Black-spotted Cuscus)*; while endangered species that are found on very high susceptible islands are *Brachylophus fasciatus (Fiji banded iguana), Emballonura semicaudata (Pacific Sheath-tailed Bat), Emoia adspersa (Micronesian Skink), Emoia boettgeri (Micronesia Forest Skink), Emoia lawesi (Olive Small-scaled Skink), Emoia trossula (Viti Barred Treeskink), Perochirus ateles (Micronesia Saw-tailed Gecko)* and *Pteropus mariannus (Marianas Flying Fox)*.

**Table 2:** Breakdown of distribution of species on different island susceptibility rankings.

Islands susceptibility	Number of islands with ≥1 species	Total number of species occurrences	Species vulnerability		
			Vulnerable (Types of Sp/number of occurrences)	Endangered (Types of Sp/number of occurrences)	C-Endangered (Types of Sp/number of occurrences)
Very high	59	114	3/5	8/107	1/2
High	178	337	8/74	12/245	6/18
Moderate	152	306	11/103	13/195	2/8
Low	171	371	35/128	44/180	23/63
Very low	114	317	36/120	35/160	19/37

Table 3 shows the number of unique species found on one island susceptibility class only and the number of islands in that susceptibility class hosting the species. The breakdown shows that islands with very high and high susceptibility ranking do not host any species that are unique to these islands only. Of course these islands are home to many species (38; see Table 2), but all these species are also found on other islands with lower susceptibility rankings. The low and very low susceptible islands host many more unique species; for example islands with very low susceptibility ranking host 14 critically endangered species that are found on these islands only and there are 16 islands across which these 14 species are distributed.

**Table 3:** The number of unique species (found on one island susceptibility class only) and the number of islands in that susceptibility class hosting the species.

Island Susceptibility	Number of unique species/Number of islands		
	Vulnerable	Endangered	Critically Endangered
Very High	0	0	0
High	0	0	0
Moderate	0	1/1	0
Low	14/17	24/28	18/19
Very Low	12/16	14/21	14/16

Species that are found on one island only are generally at a higher risk of extinction than those that are found on multiple islands, however the island size and resources available are important criteria and the above statement will only be true for similar islands. For instance, *Perochirus Ateles (Micronesia Saw-tailed Gecko)* was found on 169 islands, therefore is under a much lower risk of extinction than many other species that are found on one or only a few islands. Of the 38 critically endangered species in this study, 30 were found on one island only, while of the 61 endangered species 31 were on one island only. Fortunately, none of these islands were in the high or very high susceptible ranking, with only one island in the moderate ranking category. The list of these species and island susceptibility rankings are provided in Supplementary Table S2.

Of the 150 vulnerable, endangered and critically endangered species, 84 are endemic to this region (Table S3). These species are only found in this region of the world, and so deserve extra attention since a loss of any of these species will mean global extinction. 54 of the 84 endemic species are found on one island only, 11 are found on two islands, while the remaining 19 are found on three or more islands. Some of these species are very widespread, such as *Emoia boettgeri (Micronesia Forest Skink)* which is found on 125 islands, *Emoia tuitarere (type of lizard)* found on 127 islands and *Loveridgelaps elapoides (Solomons Black-banded krait)* found on 81 islands. Of the 54 endemic species found on one island only, 18 are critically endangered (Table S3). 16 of these are only found on La Grande Terre island in New Caledonia (area 1628 sq km), one on New Guinea in PNG (area 786000 sq km), and one on Goodenough Island in PNG (area 687 sq km). *Aproteles bulmerae* (Bulmer’s Fruit Bat) is found on New Guinea island in PNG, and while the island is very large in size, the species has a very restricted range, with the area of occupancy being less than 10 square kilometres.

**Figure 4:**
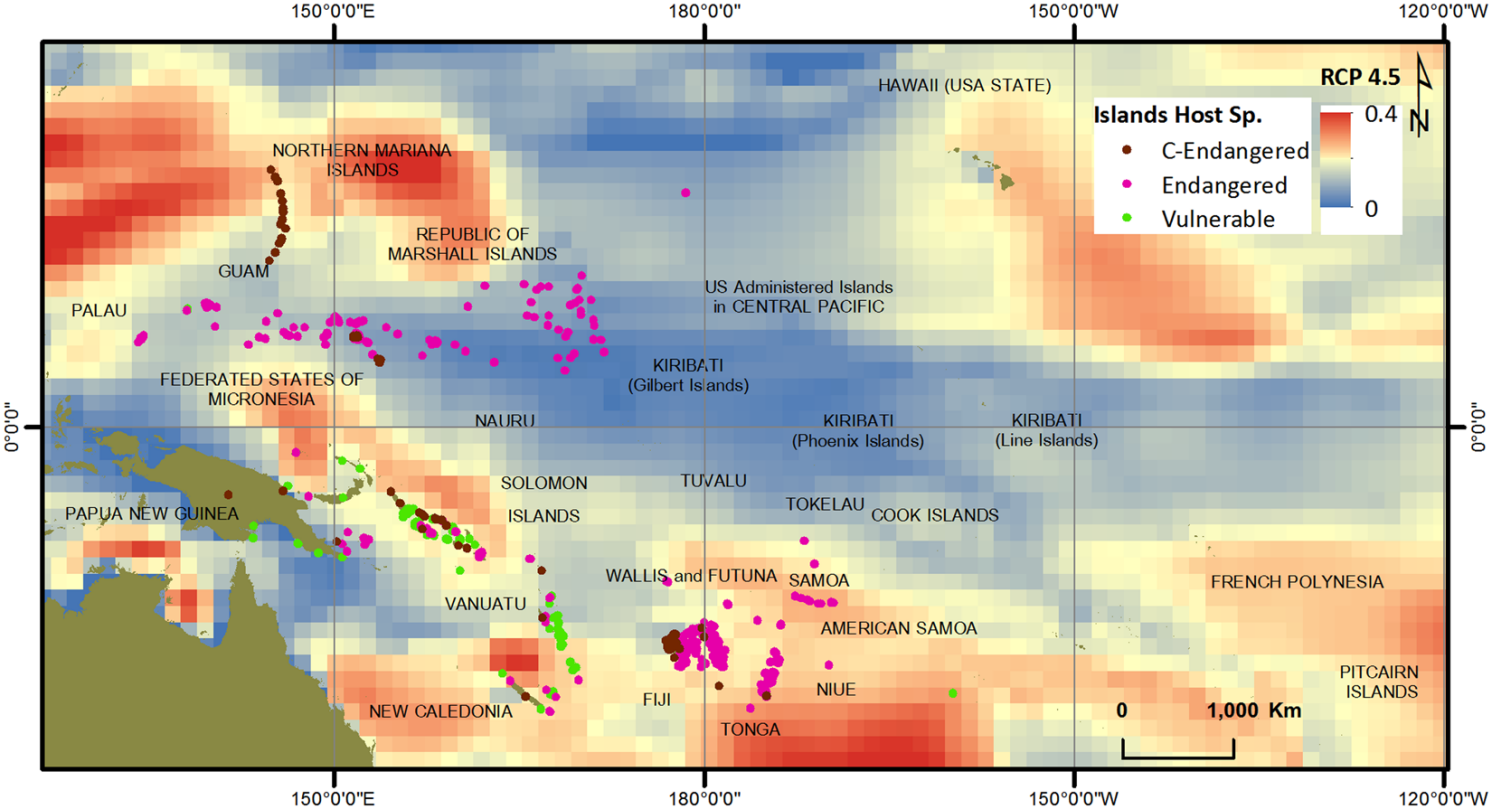
Critically endangered, endangered and vulnerable species on RCP4.5 Hs projections. (Figure generated using ARCGIS 10.3, http://www.esri.com/).

**Figure 5:**
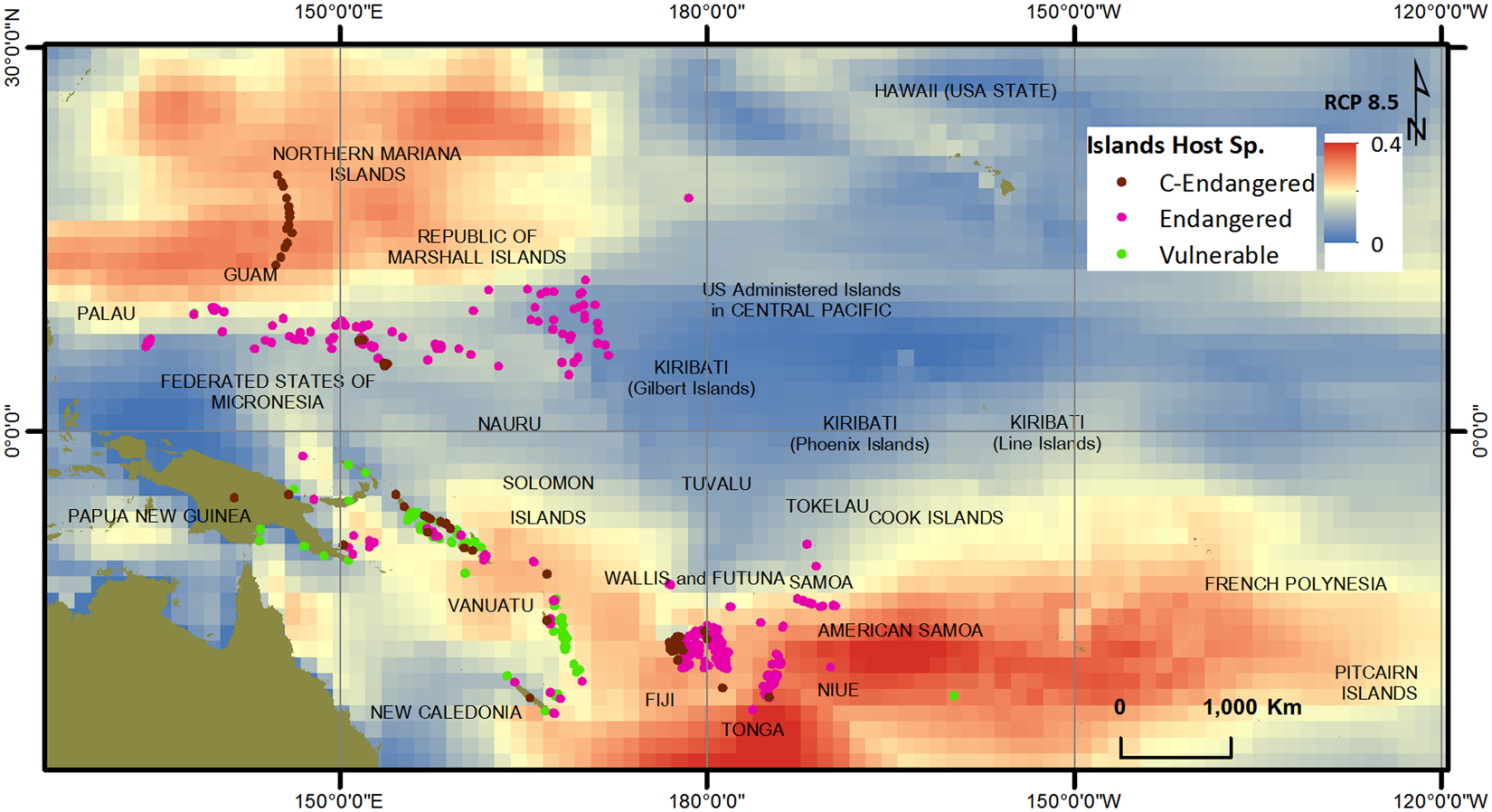
Critically endangered, endangered and vulnerable species on RCP8.5 Hs projections. (Figure generated using ARCGIS 10.3, http://www.esri.com/).

Finally, spatial data layers of critically endangered, endangered and vulnerable species were overlain on regional scale sea level rise projected mean significant wave height information. Figures 4 and 5 represent RCP 4.5 and RCP 8.5 projections with species data respectively. As can be seen from both figures, some of the Islands that host critically endangered species are located in areas with high projected wave heights, making them even more vulnerable to extinction. For analysis, the projected wave heights were divided into four categories; Low: 0.0–0.1 m, Moderate: 0.1–0.2 m, High: 0.2–0.3 m, and Very High: 0.3–0.4 m (see Figures S1 and S2). Hs values ranged from 0.0 to 0.3 m for RCP 4.5 projections and 0.0–0.4 m for RCP 8.5 projections. Under RCP 4.5 projections, most of the islands that host a critically endangered, endangered or vulnerable species are in the 0.1–0.2 m category (478/674); however under RCP 8.5 there are more islands in the higher 0.2–0.3 m category (293/674). Under RCP 4.5 only one island that is host to one of these species falls in the 0.2–0.3 m category, with no islands in the highest 0.3–0.4 m category; however under RCP 8.5 there are 293 islands in the 0.2–0.3 m category and 24 islands in the highest 0.3–0.4 m category. 23 islands with endangered species and one island with critically endangered species falls in this critical bracket (see Table S4). Of concern is the observation that large numbers of islands hosting critically endangered species fall in the higher projected Hs categories, and more so under RCP 8.5 projections. The Northern Mariana Islands, islands in Fiji, Tonga and New Caledonia host most of the critically endangered species, and Fiji, Tonga and the Northern Mariana Islands all fall in the high and very high categories under RCP 8.5 projections. Comparing the projected Hs values with the island susceptibility rankings, we see that most of the islands in the Federated States of Micronesia, Marshall Islands, Tonga and Tokelau have the highest susceptibility rankings (Fig. 1), are host to many endangered species (Fig. 4) and fall in the moderate to high projected Hs values (Figures S1 and S2), hence the species on these islands are perhaps most at risk and many of these species may need to be moved from the endangered to the critically endangered threat level.

## Conclusion

The islands in the Pacific are predominantly small in size, limestone or reefal in origin, and with low elevations. In fact the median area of the 1779 islands in our database was 1.34 sq km and 36% of the islands were reef islands, which had a maximum elevation of 3 m. The combination of low elevations, small land area, unconsolidated sediments as lithologic origins, and exposure to open oceans makes many of these islands quite vulnerable. Projected increases in sea level rise and associated mean significant wave heights, together with more intense tropical cyclones, are likely to exacerbate these vulnerabilities and result in significant habitat destruction. This will be a major threat to species inhabiting these islands, especially since habitat loss on these islands cannot be countered by dispersal^16^. The impacts will be variable for different species, depending on which type of island they inhabit, the distribution of these species, and the projected impacts of climate change. Each of the islands has unique physical and geographical attributes, and is subject to meteorologic and oceanographic conditions peculiar to its tectonic setting, hence sea level rise and projected wave height impacts will be different for different regions (countries)^17^. Species found on one or only a few islands only will generally be more vulnerable, species found on higher susceptible islands will be more vulnerable, and species found in regions with higher projected sea level rise and mean significant wave heights will be more vulnerable. For species on small islands, migration to other islands in search of suitable habitat is not an option. Extinction of endemic species found on these islands, especially if they occur on only one or a few islands, is a real possibility and could accelerate under climate change forecast by the latest IPCC report^18^.

This study has identified a number of species that face increased threats by virtue of the islands they inhabit and the impact of climate change on these islands. Of particular concern is the large number of endemic species that inhabit one island only. A total of 150 terrestrial vertebrate species classed as vulnerable, endangered or critically endangered by the IUCN inhabit 674 of the 1779 islands in the Pacific. Critically endangered species number 38, and of these 19 are endemic to the Pacific; of these 18 occur on one island only. Projected mean significant wave height data shows that many of the islands that host these species will face substantially increased threats from climate change related events in the future.

This is the first known work that relates species threat levels, as reported by the IUCN, with island susceptibility under future climate. The methodology applied here can be used for prioritization of resources and species for protection. It highlights the fact that not only the species and their threat levels are important, but also the islands or surroundings where they live. This is especially true for species found on small islands, where the opportunity to translocate is minimal, and so the state or vulnerability of the island takes added importance.

## Electronic supplementary material

Supplementary Information

## References

[1] Barnett J (2001). Adapting to climate change in Pacific Island countries: the problem of uncertainty. World Dev.

[2] Kumar L, Taylor S (2015). Exposure of coastal built assets in the South Pacific to climate risks. Nat Clim Change.

[3] Hughes C, Eastwood R (2006). Island radiation on a continental scale: exceptional rates of plant diversification after uplift of the Andes. Proceedings of the National Academy of Sciences.

[4] Kerr JT, Dobrowski SZ (2013). Predicting the impacts of global change on species, communities and ecosystems: it takes time. Global Ecology and Biogeography.

[5] Webber BL, Scott JK (2012). Rapid global change: implications for defining natives and aliens. Global Ecology and Biogeography.

[6] Kier G (2009). A global assessment of endemism and species richness across island and mainland regions. Proceedings of the National Academy of Sciences.

[7] Gillespie RG, Claridge EM, Roderick GK (2008). Biodiversity dynamics in isolated island communities: interaction between natural and human‐mediated processes. Mol Ecol.

[8] Whittaker, R. J. & Fernández-Palacios, J. M. *Island biogeography: ecology, evolution, and conservation*. (Oxford University Press, 2007).

[9] Gillespie RG, Roderick GK (2002). Arthropods on islands: colonization, speciation, and conservation. Annu Rev Entomol.

[10] Frankham, R. Do island populations have less genetic variation than mainland populations? *Heredity* **78** (1997). 10.1038/hdy.1997.46 9119706

[11] Frankham R (1998). Inbreeding and extinction: island populations. Conserv Biol.

[12] Wetzel FT, Beissmann H, Penn DJ, Jetz W (2013). Vulnerability of terrestrial island vertebrates to projected sea‐level rise. Global Change Biol.

[13] Rolland J, Salamin N (2016). Niche width impacts vertebrate diversification. Global Ecology and Biogeography.

[14] IUCN. *Red list spatial data*, http://www.iucnredlist.org/technical-documents/spatialdata (2016). Date of access: 20/08/2016.

[15] Nunn, P., Kumar, L., Eliot, I. & McLean, R. F. Regional coastal susceptibility assessment for the Pacific Islands: Technical Report. Australian Government and Australian Aid, Canberra. 123 (2015).

[16] Wetzel FT, Kissling WD, Beissmann H, Penn DJ (2012). Future climate change driven sea‐level rise: secondary consequences from human displacement for island biodiversity. Global Change Biol.

[17] Australian Bureau of Meteorology and CSIRO. Climate Variability, Extremes and Change in the Western Tropical Pacific: New Science and Updated Country Reports., Australian Bureau of Meteorology and Commonwealth Scientific and Industrial Research Organisation, Melbourne 2014.

[18] Stocker, T. *et al*. IPCC, 2013: summary for policymakers in climate change 2013: the physical science basis, contribution of working group I to the fifth assessment report of the intergovernmental panel on climate change. (Cambridge University Press, Cambridge, New York, USA, 2013).

