# Supplementary material for: Climate change impacts on the threatened terrestrial vertebrates of the Pacific Islands
Source: Sci Rep. 2017 Jul 13;7:5030. doi: 10.1038/s41598-017-05034-4 (PMC5509733; doi:10.1038/s41598-017-05034-4)
Supplement: Supplementary file 1 — Supplementary Information [file 41598_2017_5034_MOESM1_ESM.pdf]

Supplementary Information

Climate change impacts on the threatened terrestrial vertebrates of the Pacific Islands

Lalit Kumar[1,*], Mahyat Shafapour Tehrany[1]

[1]Ecosystem Management, School of Environmental and Rural Science, University of New England, Armidale, NSW 2351, Australia

* Corresponding author: lkumar@une.edu.au

Supplementary Table 1. Islands that host six or more species.

	Islands	Lithology	Area (Km^2)	Island susceptibility ranking	Number of species
**1**	Bougainville	Composite high island	9318	L	9
**2**	Buka	Composite high island	936	VL	7
**3**	Choiseul	Composite high island	3400	L	8
**4**	Guadalcanal	Composite high island	5302	VL	8
**5**	Ile des Pins (Kunie)	Continental island	152	VL	8
**6**	Kadavu	Volcanic high island	408	VL	6
**7**	Kotomo (Koutoumo)	Continental island	16	L	6
**8**	New Caledonia (La Grande Terre)	Continental island	1628	L	53
**9**	New Guinea	Composite high island	786000	VL	31
**10**	Ovalau	Volcanic high island	103	VL	7
**11**	Santa Isabel	Composite high island	3490	L	6
**12**	Savai'i	Volcanic high island	1694	VL	6
**13**	Taveuni	Volcanic high island	435	VL	8
**14**	Vanua Levu	Volcanic high island	5534	VL	6
**15**	Viti Levu	Composite high island	10388	L	10

Supplementary Table 2. List of critically endangered and endangered species found on one island only.

Critically Endangered Species	Island Susceptibly	Endangered Species	Island Susceptibly
*Aproteles bulmerae** (Bulmer's Fruit Bat)	VL	*Bavayia exsuccida** (Sclerophyll Bavayia)	L
Choerophryne siegfriedi (species of frog)	VL	*Bavayia goroensis** (Species of lizard)	L
Dendrolagus pulcherrimus (Golden-mantled Tree Kangaroo)	VL	*Bavayia ornata** (Ornate Bavayia)	L
Dendrolagus scottae (Tenkile)	VL	*Caledoniscincus auratus** (Koumac Litter Skink)	L
*Dierogekko inexpectatus** (Key New Caledonian Gecko)	L	*Caledoniscincus chazeaui** (Chazeau's Litter Skink)	L
*Dierogekko kaalaensis** (Kaala Striped Gecko)	L	*Caledoniscincus orestes** (Panié Litter Skink)	L
*Dierogekko koniambo** (Koniambo Striped Gecko)	L	*Caledoniscincus renevieri** (Renevier's Litter Skink)	L
*Dierogekko nehoueensis** (Striped Gekko)	L	*Celatiscincus similis** (Northern Pale-hipped Skink)	L
*Dierogekko poumensis** (Poum Striped Gecko)	L	*Dactylopsila tatei** (Tate's Triok)	VL
*Dierogekko thomaswhitei** (Taom Striped Gecko)	L	*Dendrolagus goodfellowi** (Goodfellow's Tree Kangaroo)	VL
*Dorcopsis atrata** (Black Dorcopsis)	VL	*Dendrolagus notatus** (Ifola tree-kangaroo)	VL
*Eurydactylodes occidentalis** (Species of lizard)	L	*Dierogekko validiclavis** (Bold-striped Gecko)	L
*Geoscincus haraldmeieri** (Meier's Skink)	L	*Echymipera davidi** (David's Echymipera)	M
Leiolopisma alazon# (Ono-i-Lau Ground Skink)	L	*Emoia aneityumensis* (Anatom Emo Skink)	VL
*Lioscincus vivae** (Species of lizard)	L	*Emoia campbelli* (Vitilevu Mountain Treeskink)	L
*Marmorosphax kaala** (Species of lizard)	L	*Eurydactylodes symmetricus* (Large-scaled chameleon gecko)	L
*Marmorosphax taom** (Species of lizard)	L	*Kanakysaurus viviparous** (Species of lizard)	L
Mirimiri acrodonta (Fijian Monkey-faced Bat)	VL	*Kanakysaurus zebratus** (Species of lizard)	L
*Nannoscincus exos** (Northern Dwarf Skink)	L	*Lioscincus maruia** (Maruia Maquis Skink)	L
*Nannoscincus hanchisteus** (Pindai Dwarf Skink)	L	*Lioscincus steindachneri** (White-lipped Forest Skink)	L
*Nannoscincus manautei**	L	*Melomys matambuai**	L

(Species of lizard)		(Manus Melomys)	
*Nyctophilus nebulosus** (New Caledonia Long-eared Bat)	L	*Nannoscincus garrulus** (Species of snake)	L
*Oedodera marmorata** (Marbled Gecko)	L	*Nannoscincus greeri** (Greer's Dwarf Skink)	L
Petaurus abidi (Northern Glider)	VL	*Nannoscincus humectus** (Forêt Plate Dwarf Skink)	L
Phalanger matanim (Telefomin Cuscus)	VL	*Nannoscincus slevini** (Slevin's Dwarf Skink)	L
Pharotis Imogene (Thomas's Big-eared Bat)	VL	*Paraleptomys rufilatus* (Northern Hydromyine)	VL
Pteralopex pulchra (Montane Monkey-faced Bat)	VL	*Paramelomys gressitti* (Gressitt's Paramelomys)	VL
Pteropus tuberculatus (Vanikoro Flying Fox)	VL	*Peroryctes broadbenti** (Giant Bandicoot)	VL
Zaglossus bartoni (Eastern Long-beaked Echidna)	VL	*Rattus vandeuseni* (Van Deusen's Rat)	VL
		Thylogale calabyi (Calaby's Pademelon)	VL
		*Thylogale lanatus** (Mountain Pademelon)	VL

Supplementary Table 3. List of Endemic species found in the study area, together with their threat levels, number and susceptibility of islands on which they are found, and RCP4.5 Hs projected values (2081-2100).

Scientific Name	Class	Red List Category†	Islands where these are found	RCP 4.5*
Aproteles bulmerae (Bulmer's Fruit Bat)	Mammal	CE	1 VL	1
Bavayia exsuccida (Sclerophyll Bavayia)	Reptile	E	1 L	2
Bavayia goroensis (Species of lizard)	Reptile	E	1 L	2
Bavayia ornate (Ornate Bavayia)	Reptile	E	1 L	2
Caledoniscincus auratus (Koumac Litter Skink)	Reptile	E	1 L	2
Caledoniscincus chazeaui (Chazeau's Litter Skink)	Reptile	E	1 L	2
Caledoniscincus orestes (Panié Litter Skink)	Reptile	E	1 L	2
Caledoniscincus renevieri (Renevier's Litter Skink)	Reptile	E	1 L	2
Caledoniscincus terma (Mandjélia litter skink)	Reptile	V	1 L	2
Celatiscincus euryotis (Southern pale-hipped skink)	Reptile	E	1 L, 1 VL	
Celatiscincus similis (Northern Pale-hipped Skink)	Reptile	E	1 L	2
Chalinolobus neocaledonicus (New Caledonia Wattled Bat)	Mammal	E	2 L	
Dactylopsila tatei (Tate's Triok)	Mammal	E	1 VL	1
Dendrolagus dorianus (Doria's Tree Kangaroo)	Mammal	V	1 VL	1
Dendrolagus goodfellowi (Goodfellow's Tree Kangaroo)	Mammal	E	1 VL	1

Dendrolagus notatus (Ifola tree-kangaroo)	Mammal	E	1 VL	1
Dendrolagus stellarum (Seri's Tree Kangaroo)	Mammal	V	1 VL	1
Dierogekko inexpectatus (Species of lizard, Key New Caledonian Gecko)	Reptile	CE	1 L	2
Dierogekko kaalaensis (Kaala Striped Gecko)	Reptile	CE	1 L	2
Dierogekko koniambo (Koniambo Striped Gecko)	Reptile	CE	1 L	2
Dierogekko nehoueensis (Striped Gekko)	Reptile	CE	1 L	2
Dierogekko poumensis (Poum Striped Gecko)	Reptile	CE	1 L	2
Dierogekko thomaswhitei (Taom Striped Gecko)	Reptile	CE	1 L	2
Dierogekko validiclavis (Bold-striped Gecko)	Reptile	E	1 L	2
Dorcopsis atrata (Black Dorcopsis)	Mammal	CE	1 VL	1
Echymipera davidi (David's Echymipera)	Mammal	E	1 M	1
Emoia boettgeri (Micronesia Forest Skink)	Reptile	E	8 VL, 15 L, 9 M, 49 H, 44 VH	
Emoia erronan (Erronan Treeskink)	Reptile	V	1 M, 1 VL	
Emoia loyaltiensis (Loyalty Islands Emoia)	Reptile	V	4 M, 3 L	
Emoia mokosariniveikau (Vanualevu Slender Treeskink)	Reptile	E	1 L, 3 VL	
Emoia parkeri (Viti Copper-headed Skink)	Reptile	V	5 VL, 3 L, 2 M	
Emoia tuitarere (species of lizard)	Reptile	V	33 H, 14 L, 57 M, 3 VH, 20 VL	
Eurydactylodes occidentalis (Species of lizard)	Reptile	CE	1 L	2
Geoscincus haraldmeieri (Meier's Skink)	Reptile	CE	1 L	2
Graciliscincus shonae	Reptile	V	1 L, 1 VL	

(Gracile burrowing skink)				
Kanakysaurus viviparous (Species of lizard)	Reptile	E	1 L	2
Kanakysaurus zebratus (Species of lizard)	Reptile	E	1 L	2
Lacertoides pardalis (New Caledonian Leopard Skink)	Reptile	V	1 L	2
Lepidodactylus euaensis ('Eua Forest Gecko)	Reptile	CE	2 L	
Lepidodactylus manni (Viti Forest Gecko)	Reptile	V	19 H, 16 L, 15 M, 15 VL	
Lioscincus maruia (Maruia Maquis Skink)	Reptile	E	1 L	2
Lioscincus steindachneri (White-lipped Forest Skink)	Reptile	E	1 L	2
Lioscincus vivae (Species of lizard)	Reptile	CE	1 L	2
Loveridgelaps elapoides (Solomons Black-banded krait)	Reptile	V	13 H, 37 L, 17 M, 14 VL	
Marmorosphax boulinda (Species of lizard)	Reptile	V	1 L	2
Marmorosphax kaala (Species of lizard)	Reptile	CE	1 L	2
Marmorosphax Montana (Montane Marble-throated Skink)	Reptile	V	1 L	2
Marmorosphax taom (Species of lizard)	Reptile	CE	1 L	2
Melomys matambuai (Manus Melomys)	Mammal	E	1 L	3
Murexia rothschildi (Broad-striped Dasyure)	Mammal	V	1 VL	1
Nannoscincus exos (Northern Dwarf Skink)	Reptile	CE	1 L	2
Nannoscincus garrulous (Species of snake)	Reptile	E	1 L	2
Nannoscincus gracilis (Gracile Dwarf Skink)	Reptile	V	1 L	2

Nannoscincus greeri (Greer's Dwarf Skink)	Reptile	E	1 L	2
Nannoscincus hanchisteus (Pindai Dwarf Skink)	Reptile	CE	1 L	2
Nannoscincus humectus (Forêt Plate Dwarf Skink)	Reptile	E	1 L	2
Nannoscincus manautei (Species of lizard)	Reptile	CE	1 L	2
Nannoscincus mariei (Earless Dwarf Skink)	Reptile	V	1 L, 1 VL	
Nannoscincus rankini (Rankin's Dwarf Skink)	Reptile	V	1 L	2
Nannoscincus slevini (Slevin's Dwarf Skink)	Reptile	E	1 L	2
Nyctophilus nebulosus (New Caledonia Long-eared Bat)	Mammal	CE	1 L	2
Oedodera marmorata (Marbled Gecko)	Reptile	CE	1 L	2
Ogmodon vitianus (Fiji Snake)	Reptile	E	9 H, 9 M, 6 L, 1 VL	
Peroryctes broadbenti (Giant Bandicoot)	Mammal	E	1 VL	1
Phoboscincus bocourti (Bocourt's Terrific Skink)	Reptile	E	1 L, 1 VL	
Pogonomys fergussoniensis (D'entrecasteaux Archipelago Pogonomys)	Mammal	E	3 VL	
Pteropus anetianus (Vanauatu Flying Fox)	Mammal	V	3 L, 16 VL, 1 M	
Pteropus cognatus (Makira Flying Fox)	Mammal	E	3 L, 1 VL, 1 M	
Pteropus molossinus (Pohnpei Flying Fox)	Mammal	V	1 VL, 1 VH	
Pteropus ornatus (Ornate Flying Fox)	Mammal	V	3 L, 1 VL	
Pteropus rennelli (Rennell Flying Fox)	Mammal	V	1 L	2
Pteropus vetulus (New Caledonia Flying Fox)	Mammal	V	1 L, 1 VL	
Pteropus woodfordi	Mammal	V	5 L, 5 VL, 1 H, 1 VH	

(Dwarf Flying Fox)				
Pteropus yapensis<br>(Yap flying fox)	Mammal	V	4 L	
Rhacodactylus chahoua<br>(Bavay's Giant Gecko)	Reptile	V	3 L, 1 VL	
Rhacodactylus ciliates<br>(Eyelash Gecko)	Reptile	V	2 L, 2 VL	
Rhacodactylus sarasinorum<br>(Sarasin's Giant Gecko)	Reptile	V	1 L	2
Rhacodactylus trachyrhynchus<br>(Rough-snouted Giant Gecko)	Reptile	E	2 L, 1 VL	
Sigaloseps ruficauda<br>(Red-tailed Shiny Skink)	Reptile	V	1 L	2
Simiscincus aurantiacus<br>(Orange-bellied Burrowing Skink)	Reptile	V	1 L, 1 VL	
Solomys sapientis<br>(Isabel Giant Rat)	Mammal	E	2 L, 1 M, 2 H	
Thylogale lanatus<br>(Mountain Pademelon)	Mammal	E	1 VL	1
Tropidoscincus aubrianus<br>(Aubrey's Whiptailed Skink)	Reptile	V	3 L, 1 VL	
Uromys rex<br>(King Rat)	Mammal	E	2 VL	

*1=0-0.1m, 2=0.1-0.2m, 3=0.2-0.3m, 4=0.3-0.4m.

† CE=Critically Endangered, E=Endangered, V=Vulnerable

Supplementary Table 4. Number of islands in different categories under RCP 4.5 and RCP 8.5 projected Hs values (2081-2100).

	**RCP 4.5 Projected Hs Values**			
	0-0.1 m	0.1-0.2 m	0.2-0.3 m	0.3-0.4 m
C-Endangered	22	78	0	0
Endangered	160	302	1	0
Vulnerable	13	98	0	0
	**RCP 8.5 Projected Hs Values**			
C-Endangered	14	65	36	1
Endangered	60	146	252	23
Vulnerable	51	44	5	0

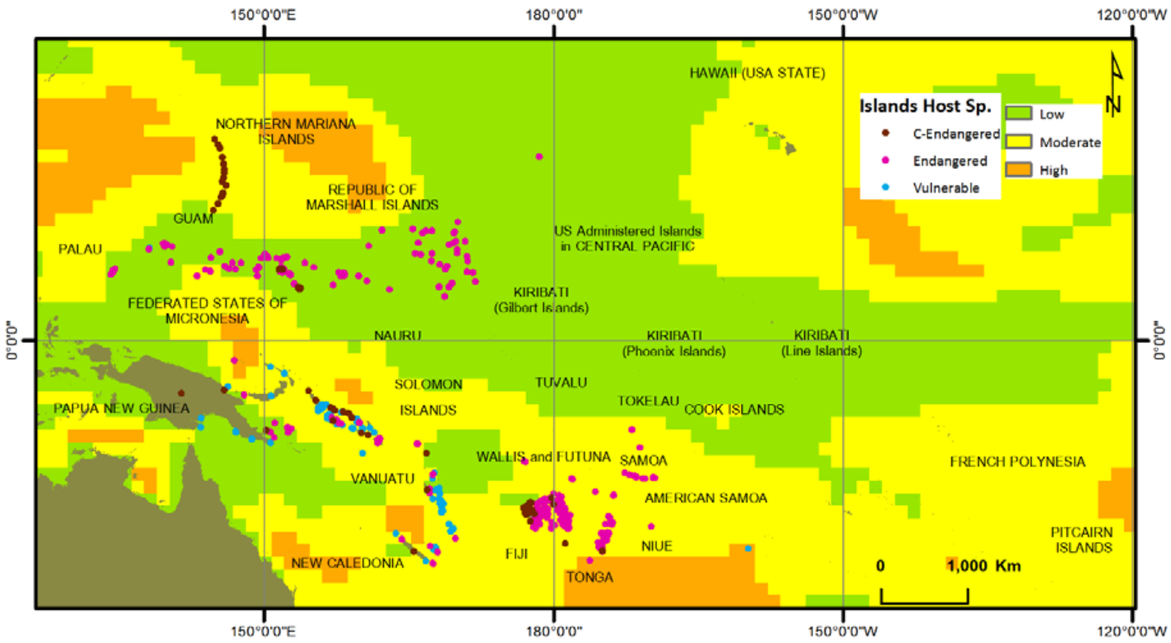

Figure S1 RCP 4.5 Projected Hs values for 2081-2100 reclassified into four categories. (Figure generated using ARCGIS 10.3, http://www.esri.com/). The concentration pathway RCP 4.5 (a medium low emission scenario) was used under the CMIP5 (Coupled Model Intercomparison Project Phase 5) model. The climate models used were CNRM-CM5, HadGEM2-ES, INMCM4 and ACCESS1.0.

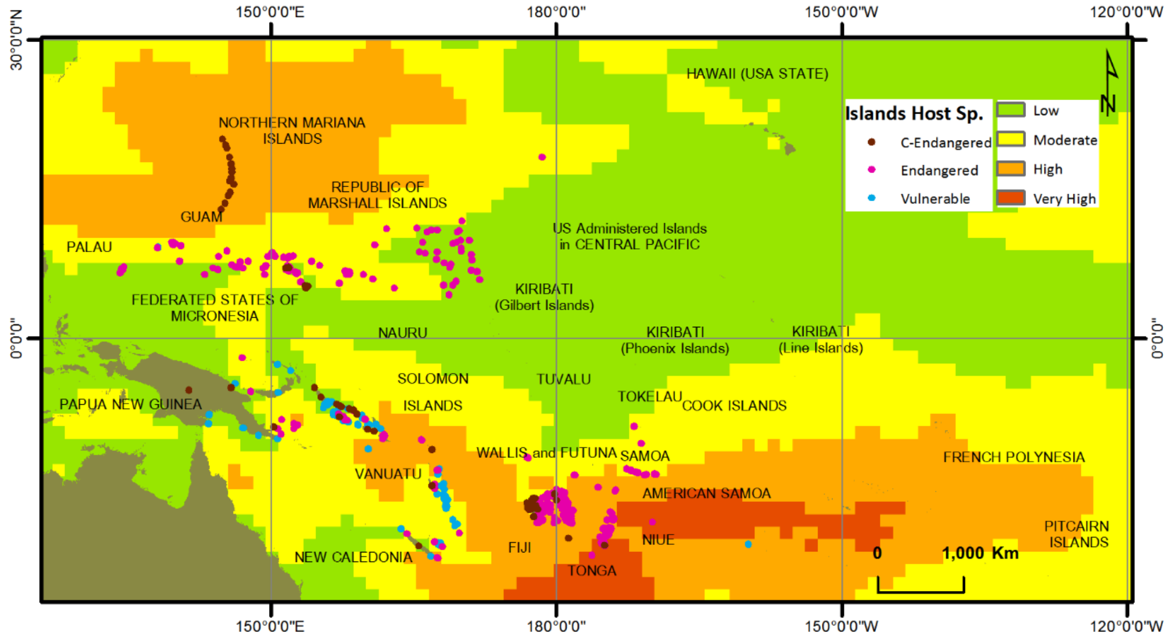

Figure S2. RCP 8.5 Projected Hs values for 2081-2100 reclassified into four categories. (Figure generated using ARCGIS 10.3, http://www.esri.com/). The concentration pathway RCP 8.5 (a high emission scenario) was used under the CMIP5 (Coupled Model Intercomparison Project Phase 5) model. The climate models used were CNRM-CM5, HadGEM2-ES, INMCM4 and ACCESS1.0.
